# Approach to Cystic Lesions of the Pancreas: Review of Literature

**DOI:** 10.7759/cureus.36827

**Published:** 2023-03-28

**Authors:** Amit Gupta, Jaine J Chennatt, Chirag Mandal, Jitendra Gupta, Shyam Krishnasamy, Bodhisattva Bose, Pratik Solanki, Sunil H, Sunil Kumar Singh, Sweety Gupta

**Affiliations:** 1 Surgery, All India Institute of Medical Sciences, Rishikesh, Rishikesh, IND; 2 Radiology/Interventional Radiology, All India Institute of Medical Sciences, Rishikesh, Rishikesh, IND; 3 Surgical Oncology, All India Institute of Medical Sciences, Rishikesh, Rishikesh, IND; 4 Radiation Oncology, All India Institute of Medical Sciences, Rishikesh, Rishikesh, IND

**Keywords:** cystic, pseudocyst, resection, pancreatoduodenectomy, pancreas

## Abstract

Pancreatic cystic lesions (PCL) have a wide range of demographical, clinical, morphological and histological characteristics. The distinction between these lesions is of paramount importance due to the risk of malignancy in specific categories of PCL. Considering the malignant potential for pancreatic cystic neoplasm (PCN) lesions, guidelines have been made to balance unnecessary treatment and manage the progression to malignancy. Various surgical procedures can be done for PCN depending on the location and size of the cyst; pancreatoduodenectomy is done for PCN located in the head of the uncinate process, whereas distal pancreatectomy is done for PCN in the body or tail. In the neck and proximal body of the pancreas, less extensive resections such as central pancreatectomy can be performed. Active surveillance of PCN is typically offered to asymptomatic PCNs of subtype intraductal papillary mucinous neoplasms (IPMN) and mucinous cystic neoplasms (MCN) without any concerning features. In recent years, numerous guidelines have been created to augment PCN diagnosis, classification and management. Despite this, the management of PCNs remains complex. Thus, discussions with multidisciplinary teams involving surgeons, gastroenterologists, pathologists, and radiologists are required to ensure optimum care for the patient.

## Introduction and background

Pancreatic cystic lesions (PCL) are a comprehensive group of pancreatic pathology and are broadly classified into non-neoplastic lesions and cystic neoplasms. These lesions have a wide range of demographical, clinical, morphological and histological characteristics [1]. The incidence and prevalence of PCLs have been steadily increasing in recent years, primarily due to clinician awareness and exponential use and advancement of radiology in the detection of these lesions. The distinction between these lesions is of paramount importance due to the risk of malignancy in specific categories of PCL. Differentiating between the different types of PCL can be challenging due to the similar imaging characteristics of the subtypes of PCL. However, a combined approach of cross-sectional imaging, endoscopic ultrasound (EUS) +/- fine needle aspiration (FNA) with cytological and biochemical analysis almost certainly clinches the diagnosis. Thus, this review article discusses the various approaches to PCL with emphasis on pancreatic pseudocyst and pancreatic cystic neoplasms (PCN), their classification, diagnosis, guidelines, and subsequent management [2].

## Review

Classification

Until recently, the spectrum of PCL was restricted to mucinous and serous neoplasms. However, since the 1980s, there has been an exponential advancement in radiology and other diagnostic techniques, leading to a drastic increase in awareness and subsequent research on PCLs. Thereafter, new classification systems were created, leading to more studies on the pathogenesis, morphology, clinical spectrum, and management of these new entities of PCL [3]. Currently, PCLs are classified broadly into non-neoplastic and neoplastic cysts, and subdivided into epithelial and nonepithelial cysts (Table 1).

**Table 1:** Kloppel's Classification of Pancreatic Cystic Lesions MCN-Mucinous cystic neoplasm IPMN- Intraductal Papillary Mucinous Neoplasms [3] “-“Not applicable

Neoplastic		Non-neoplastic	
Epithelial		Epithelial	
Benign		Benign	
	Serous adenoma (microcystic)		Lymphoepithelial cyst
	Serous adenoma (oligocystic, ill-demarcated)		Congenital cyst (in malformation syndromes)
	MCN		Mucinous nonneoplastic cyst
	IPMN		Enterogeneous cyst
	Acinar cell cystadenoma		Retention cyst
	Dermoid cyst		Periampullary duodenal wall cyst
	Cystic hamartoma		Endometrial cyst
	von Hippel-Lindau–associated cystic neoplasm		-
Borderline		-	-
	MCN		
	IPMN		
	Solid pseudopapillary tumor		
Malignant		-	-
	MCN-associated carcinoma		
	IPMN-associated carcinoma		
	Ductal adenocarcinoma, cystic		
	Serous cystadenocarcinoma		
	Pancreatoblastoma, cystic		
	Cystic metastatic epithelial neoplasm		
	Neuroendocrine carcinoma, cystic		
Nonepithelial	Benign neoplasm(i.e.,lymphangioma) Malignant neoplasm (i.e.,sarcoma)	-	Pseudocyst Parasitic cyst

Non-neoplastic cysts consist of pancreatic pseudocyst, congenital cysts, lympho-epithelial cysts, mucinous non-neoplastic cysts, enterogenous cysts, retention cysts, periampullary duodenal wall cysts, and endometrial cyst. Of the following, pseudocysts are most commonly seen in clinical practice and will be discussed in detail in the following sections. Neoplastic cysts or PCN are broadly classified according to the World Health Organisation classification of pancreatic tumours (Table 2) into Benign, Borderline, and Malignant lesions [4].

**Table 2:** World Health Organization classification of pancreatic tumours [4]

Nature	Subtypes
Benign	Serous cystadenoma
	Mucinous cystadenoma
	Intraductal papillary mucinous adenoma
	Mature teratoma
Borderline	Mucinous cystic tumour with moderate dysplasia
	Intraductal mucinous papillary tumour with moderate dysplasia
	Solid pseudopapillary tumour
Malignant	Highly ductal dysplasia, carcinoma in situ
	Ductal adenocarcinoma
	Serous cystadenocarcinoma
	Mucinous cystadenocarcinoma
	Intraductal papillary mucinous carcinoma
	Acinar cell carcinoma
	Solid pseudopapillary carcinoma
	Pancreaticoblastoma
	Osteoclasts similar to giant cell tumor
	Miscellaneous carcinomas

PCNs are a heterogenous group of lesions comprising 5-15% of all PCLs [5]. The most commonly encountered lesions in clinical surgical practice include serous cystic neoplasms (SCN), mucinous cystic neoplasms (MCN) and intraductal papillary mucinous neoplasms (IPMN). 

Pseudocyst of the pancreas

Pancreatic pseudocysts are the most common PCL accounting for approximately 75% of all PCLs. These are inflammatory cysts consisting of fluid rich in pancreatic enzymes or protease-free serous fluid enclosed by a wall of fibrous or granulation tissue. They are named so because of the lack of epithelial lining. They are formed due to autodigestive tissue necrosis secondary to acute or chronic pancreatitis. Pseudocysts occur when an acute post-pancreatic fluid collection fails to resolve spontaneously and walls off; this usually occurs four weeks after the onset of acute pancreatitis. Pseudocysts may occur anywhere in the pancreas and can even be extra-pancreatic. They vary in size from 3 to 20 cm; larger sizes are associated with alcohol-induced pancreatitis and are most commonly located in the lesser omental sac. Other common locations include retroperitoneum, stomach, liver, and perirenal or subdiaphragmatic space. They may or may not communicate with the ductal system and are usually single but may be multiple in 10% of cases [5]. While most PCLs are asymptomatic, pancreatic pseudocysts may present with abdominal pain, early satiety, nausea and vomiting, jaundice, cholangitis as well as gastrointestinal bleeding depending on the location of the cyst [6]. The most important predictors of symptoms are the size and duration of illness [7]. Weight loss may be seen in 20% of patients due to gastric compression, poor oral intake, and maldigestion. Palpable fullness may be noted on examination in large pseudocysts.

**Figure 1:**
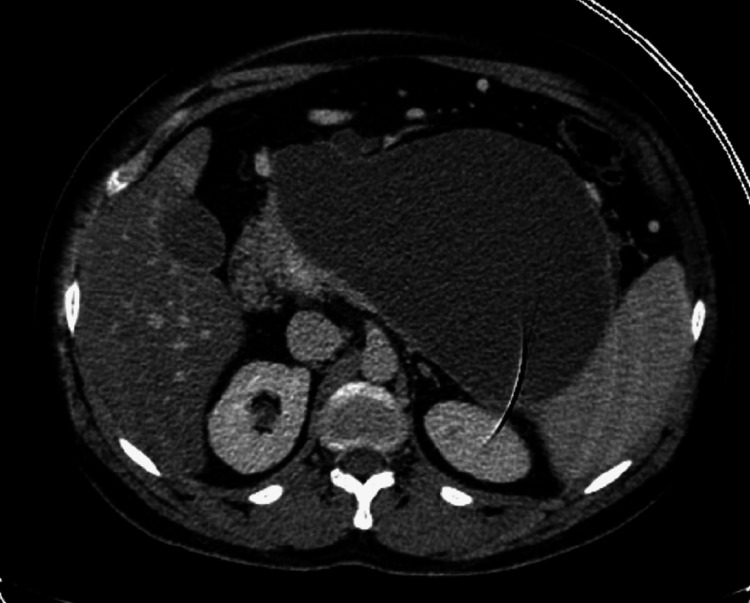
Axial section of CT scan showing pancreatic pseudocyst

The initial investigation of choice of pseudocysts is a transabdominal ultrasound (US) [8]. They are described as well-defined anechoic lesions with distal acoustic enhancement. Any suspected pseudocyst on US would require a cross-sectional imaging, mostly CT scan. On CT, they appear as round fluid-filled structures surrounded by a thick, dense wall and may be unilocular or multilocular with fibrotic strands within the cavity-causing septations (Figure 1).

High attenuation areas may be noted within the cavity, indicating the presence of debris, blood, or infections. CT scans also provide additional information about anatomy and pathology. The difficulty arises in cases where there is no evidence of pancreatitis when pseudocysts may be confused with pancreatic mucinous cysts. In these cases, cyst fluid analysis may help diagnose the lesion. Besides CT scans, MRI abdomen and magnetic resonance cholangiopancreatography (MRCP) is also used selectively in cases where communication with the biliary system cannot be accurately determined [8].

EUS can differentiate pancreatic cysts from other PCLs. In EUS, pseudocysts appear as anechoic fluid-filled structures surrounded by a thick hyperechoic rim. Debris may be noted in the dependent portion of the cavity, and colour Doppler often reveals multiple prominent vessels [9]. EUS-guided FNA with analysis of the cystic fluid can be done and can accurately differentiate between pseudocysts and neoplastic pancreatic cysts in more than 90% of patients. Table 3 describes the typical differentiating characteristics between various subtypes of PCL [9].

**Table 3:** Pancreatic cyst fluid analysis SCN- Serous cystic neoplasm MCN- Mucinous cystic neoplasm IPMN- Intraductal papillary mucinous neoplasms CEA- Carcinoembryonic antigen KRAS- Kirsten rat sarcoma virus mutation

	Pseudocyst	SCN	MCN	IPMN
CEA (Carcinoembryonic antigen)	Low(<5ng/ml)	Low ( <5ng/ml)	High (192ng/ml)	High (192ng/ml)
Cytology	No typical findings; inflammatory cells and debris may be present	Glycogen-rich cuboidal cells, (+) Periodic acid Schiff stain	Sheets and clusters of mucin-containing columnar cells with variable atypia	Tall columnar mucin-containing columnar cells with variable atypia
Mucin	Negative	Negative	Present	Present
KRAS (Kirsten rat sarcoma virus )mutation	Absent	Absent	Present	Present
Appearance and viscosity	Thin brown fluid	Thin fluid; may be bloody	Thick clear fluid specific but not sensitive	Thick clear fluid specific but not sensitive
Amylase	Very High (>250U/L)	Low	Variable – Low	Variable – High

Special note is that evidence of epithelial cells in the fluid analysis is more suggestive of a PCN than a pseudocyst. Elevated amylase levels are suggestive of a connection with the main pancreatic duct (MPD) and help with the diagnosis of a pseudocyst. Tumor markers such as carcinoembryonic antigen (CEA), on the other hand, are relatively low compared to MCN and IPMN [10].

The management of pancreatic pseudocysts includes conservative management, image-guided drainage, and surgical intervention. The majority of simple small uncomplicated pseudocysts (<4cm) are known to resolve spontaneously; hence simple observation with periodic follow-up is sufficient. However, with the increase in the cyst size, most patients become symptomatic and may develop complications. The presence of symptoms is also indication for treatment, not only presence of complications [11]. Drainage of pseudocysts can be done by US/CT guidance or EUS guidance. Percutaneous US/CT guidance with the fluid being drained into an external collection system can be performed with acceptable short-term success, but the high risk of infectious complications precludes its use in clinical practice. The presence of ductal communication with the cyst is also a contraindication [12]. Compared to USG and CT guidance, EUS-guided drainage is an excellent method for drainage of pseudocysts. It can be accompanied by a EUS-guided FNAC, especially in indeterminate cases. A transpapillary endoscopic retrograde cholangiopancreatography (ERCP) approach or a direct endoscopic transgastric or transduodenal drainage can be performed. The transpapillary approach is required when there is ductal communication, primarily if located in the head, in infected cases, or in cases with associated strictures or leaks of the MPD. Trans-gastric or trans-duodenal drainage is used when the pseudocyst lies in proximity to the gastroduodenal wall. Relative contraindications include a cyst wall thickness > 1cm or large intervening vessels/varices. EUS-guided drainage is successive 90% of the time with a recurrence rate of <10% [13].

Surgical management includes internal drainage and pseudocyst resection. Internal drainage is preferred in uncomplicated mature pseudocysts. Cystogastrostomy can be done in cases where the cysts are adherent to the posterior wall of the stomach, whereas cystoduodenostomy can be done in small cysts in the head and uncinate process of the pancreas. On the other hand, cystojejunostomy can be done for all other cysts, including large cysts or cysts more than 15cm [6].

Cystogastrostomy is a simple and fast procedure; infectious complications are less common, but it is associated with a higher incidence of upper gastrointestinal bleeding. There is no significant difference noted in terms of recurrence rate, morbidity, or mortality between cystogastrostomy and cystojejunostomy. However, cystogastrostomy was reported to have fewer complications in terms of blood loss and duration of surgery [7].

Pseudocyst resection can be done in certain cases of chronic pancreatitis, including chronic pain associated with chronic pancreatitis, multiple cysts, hemorrhage from pseudoaneurysm, obstruction, or cysts located in the uncinate process which are technically difficult to drain. Different resections can be performed depending on the location of cysts, including enucleation, partial left-sided pancreatectomy, Whipple procedure, pylorus-preserving pancreatoduodenectomy, Beger operation, and Frey procedure. Laparoscopic approaches can be done with studies showing low complication rates, good outcomes postoperatively, decreased hospital and postoperative recovery time and decreased incidence of bleeding complications [14].

Pancreatic cystic neoplasms

PCNs are a heterogenous group of cystic lesions that range from SCN, MCN, and IPMN to other rare lesions such as cystic neuroendocrine tumors (cNET) and solid pseudopapillary neoplasms (SPN). This diverse group of pancreatic cysts has varied clinical, radiological, and pathological features (Table 4) and differentiation between them is paramount due to their varied risk of malignancy [15].

**Table 4:** Common characteristics of different pancreatic cystic lesions SCN- Serous cystic neoplasm MCN- Mucinous cystic neoplasm IPMN- Intraductal papillary mucinous neoplasms CEA- Carcinoembryonic antigen KRAS- Kirsten rat sarcoma virus mutation

Parameters	Pseudocyst	IPMN	MCN	SCN
Demographics	Alcohol abuse, History of pancreatitis Middle-aged men	Middle-aged and older individuals Equal gender distribution	Variable, 5^th^ to 7^th^ decade More common in women	Variable, 5^th^ to 7^th^ decade More common in women
Age		60-70	40-50	50-70
Location	Entire pancreas Tail Solitary Variable size	Head May be incidental and multifocal	Body and tail Incidental Solitary	Entire pancreas Many small cysts/ or oligo/macrocystic
Communication with duct	Frequently	Yes	Occasionally	Rare
Computed tomography (CT)/Magnetic resonance Imaging (MRI) appearance	Unilocular cyst, parenchymal inflammatory changes	MD-IPMN: Dilated MPD or dilated PD with dilated side branches BD-IPMN: Cyst or cluster/ Grape-like cystic lesion, may be multifocal, dilated side branches	Large cysts with thick septae, peripheral calcification, wall thickening	Microcystic multiple small cysts, central fibrous scar with calcification, oligocytic
EUS findings	Thick walled, anechoic, unilocular cystic lesion, chronic pancreatitis	MD-IPMN: Dilatation of MPD, hyperechoic nodules arising ductal wall, Fish eye ampulla (Bulging ampulla extruding mucin) Bile duct(BD) IPMN: Small-cluster of grape-like dilatations of BD, mural nodule	Macrocystic lesion with few septations. Focal, peripheral calcification may be present. No ductal dilatation. Atypical papillary projections String sign positive (Drop of cyst fluid stretched between thumb and index finger, length > 3.5mm is positive for MCN)	Multiple, small, anechoic cystic areas and 'honeycomb' appearance. Central fibrosis/ calcification may be present
Confocal endomicroscopy	(-)	Epithelial villous structures, no vascular networking	Epithelial villous structures, no vascular networking	Thickened cyst wall; unilocular vascular networking; fibrous bands

Prevalence of PCN in abdominal ultrasound was noted to be 0.21%, in CT 2.6%, and in MRI with MRCP 2.4 to 49.1%. However, in autopsy studies, PCN was reported to be as high as 50% of patients. There is no gender predilection, but increasing age had a strong correlation [16].

Serous cystic neoplasms are focal well-demarcated lesions that consist of multiple small cysts (<1-2cm) with dense fibrous septations, which gives it its honeycomb appearance. A pathognomonic feature seen is sunburst calcification caused by central fibrosis or calcification. However, this is seen in only 10% of the patients. Less common variants include a macrocystic variant (>2cm cysts) and a solid variant that consists of multiple microcysts, which appear as a hypoechoic mass on CT scan. Cytology with findings of glycogen-positive staining cells established the diagnosis in 50% of the patients [17].

Mucinous cystic neoplasms are septated thin-walled fluid-filled cystic lesions. Diagnostic features include epithelial cells (cuboidal or columnar). Pathognomonic features include eccentric calcifications but are seen in only 15% of cases. Ductal communication is rare, but there is a 25% chance of malignancy which is suggested by larger sizes, solid regions, or a solid mass. Elevated CEA or Mucin is indicative of malignancy [18].

IPMNs originate from the main pancreatic duct or side branches. Characteristic features include the production of mucin, ductal dilatation, and papillary epithelial growth. A fish mouth of gaping papilla may be present. EUS findings include dilated main or side ducts with mucin and mural nodules. Imaging features on CT scan suggestive of IPMN include focal hypoechoic mass, mural nodules, or unilocular cystic lesions [19]. The different characteristics, clinical features, diagnosis, and management of PCLs will be discussed in detail in subsequent sections.

Clinical Features

The majority of PCNs are incidentally diagnosed on imaging studies; typical pancreatic symptoms are usually absent. Jaundice may be seen secondary to external compression of the common bile duct (CBD) by the PCN or a mucin plug. MD-IPMN with mucin production may be associated with acute pancreatitis as the mucin plugs may occlude the MPD causing acute pancreatitis and abdominal pain with elevated amylase levels. Endocrine/exocrine function disruption or insufficiency may also occur with progressive inflammation. Jaundice and pancreatitis may also occur in the setting of advanced neoplasia [20].

Risk of Malignancy

SCN is most commonly benign with no need for active surveillance. On the other hand, IPMN, MCN, SPN, and cNET are considered to have malignant potential, and hence surgical resection or active surveillance is required. Amongst PCN, IPMN is considered to be the most common [18]. This is of clinical significance as high-grade dysplasia, or invasive cancer, is reported in as high as 30% of patients who underwent resection for side branch (SB) IPMN. Main duct involvement is also associated with an increased risk (62%) of advanced neoplasia and conventional pancreatic ductal adenocarcinoma in other areas of the pancreas. Similarly, in resected MCN specimens, the risk of advanced neoplasia has been noted to be 10-39%, whereas invasive cancer was reported to be seen in up to 15% of cases. Invasive cancer was also seen in up to 10% of patients in cNET tumors [21,22].

Diagnosis

Differentiation between the various subtypes of PCN is the crucial step in diagnosis as the management varies according to the subtype and its related malignant potential. The typical workup of PCN includes a pancreatic protocol CT and gadolinium-enhanced MRI with MRCP and EUS [23].

MRI with MRCP is used to determine the involvement of the pancreatic duct and is more sensitive to detect mural nodules or internal septations as compared to a CT scan. MRI with MRCP is also used for follow-up studies to reduce exposure to ionizing radiation [22]. IPMN is most commonly seen in the head of the pancreas (70%), followed by the body/tail (20%) and is multifocal in 5-10% of the cases [15]. In contrast, MCN is seen most commonly in the body and tail and is unilocular or of septated macrocystic type. 

EUS is used as an adjunct if specific clinical or radiological features are present or if cytological examination or biochemical analysis is required to differentiate between the lesions. EUS can determine the cyst size, location, thickness, focal wall irregularity, mural nodule or mass, septations, debri, mucus or dilatation of MPD. The indications for EUS are as described in the American Gastroenterological Association (AGA) guidelines (2015), International Association of Pancreatology (IAP) guidelines (2017), and European guidelines (2018) and are summarized in Table 5.

**Table 5:** Indications for endoscopic ultrasound in PCL PCL- Pancreatic cystic lesion PD- Pancreatic duct EUS- Endoscopic ultrasound FNA- Fine needle aspiration CA19-9- Carcinoembryonic antigen 19-9 IPMN- Intraductal papillary mucinous neoplasms PCN- Pancreatic cystic neoplasm

Guidelines	Indications
American Gastroenterological Association(AGA) Guidelines (2015)	At least two of the following concerning features Cyst diameter >30mm Nodule Pancreatic duct dilatation
International Association of Pancreatology(IAP) Guidelines (2017)	Growth rate ≥5mm over two years Increased levels of serum CA19-9 PD dilatation between 5 and 9mm Cyst diameter ≥30mm Acute pancreatitis (caused by IPMN) Enhancing mural nodule
European guidelines (2018)	EUS–(FNA)should only be performed when the results are expected to change clinical management. EUS–(FNA) is recommended if the PCN has either clinical or radiological features of concern identified during the initial investigation or surveillance

EUS can also be augmented with FNA to get samples for cytopathological examination and other biochemical examinations to distinguish between the different subtypes of PCN (Table 3). However, cyst fluid is usually acellular and hence has limitations in the cytopathological examination. Through-the-needle forceps devices have now been introduced for EUS-guided tissue sampling. A standard 19-gauge EUS needle is used through which a micro-forceps (outer diameter <1mm) is passed and used to obtain cyst wall/mural nodule samples. However, studies are yet to be performed before its use in standard practice. EUS-FNA is a relatively safe procedure with a 2-3% complication rate. Complications include abdominal pain, infection, intra-cystic bleeding or pancreatitis. The risk of peritoneal metastases and needle-tract seeding is rare; hence EUS-FNA can be safely performed in suspected malignancy cases [24-29].

Cyst Fluid Analysis

Cyst fluid can be analyzed for tumour markers such as CEA, mucin, amylase, cytology and other molecular markers. Cyst fluid CEA is the most useful for differentiation between mucinous and non-mucinous PCN and has a higher accuracy for detection of MCN when compared to EUS morphology or cytology. CEA is used because mucinous cysts are lined by endoderm-derived columnar epithelial cells, which secrete CEA in contrast to non-mucinous cysts. Amylase is also routinely used in the biochemical analysis of cyst fluid and elevated amylase supports more the diagnosis of pseudocyst. Elevated CEA levels are indicative of a connection with the pancreatic duct; however, it can also be raised in MCN. A third marker used is fluid glucose level which is elevated in mucinous PCN. However, evidence is still lacking; hence further studies are required. Deoxyribonucleic acid (DNA) tests have also been introduced as a promising adjunct to differentiate between mucinous subtypes and premalignant and advanced lesions. DNA biomarkers such as KRAS mutations, guanine nucleotide binding protein, alpha stimulating (GNAS) mutations are sensitive and specific for IPMN. They are less sensitive for MCN but not sensitive or specific for SCN. However, the use of these tests is expensive and time-consuming and is currently not integrated into management guidelines [30-33].

Management and guideline recommendations

Considering the malignant potential for PCN lesions, guidelines for either close monitoring or management are needed to balance unnecessary treatment and manage the progression to malignancy. Five guidelines that offer recommendations on PCN surveillance and surgical resection based on the risk of malignancy are American Gastroenterology Association (2015), International Consensus Guidelines (2017), European guidelines (2018), American College Gastroenterology (2018) and Radiology White paper (2017) [34,35].

Active Surveillance of PCN

Active surveillance of PCN is typically offered to asymptomatic PCNs of subtype IPMN and MCN without any concerning features (Table 6).

**Table 6:** Worrisome and high-risk features CA19-9- Carbohydrate antigen 19-9

Category	Characteristics
Worrisome features	Diameter of >- 3cm
	Enhancing mural nodules > 5mm
	Thickened/enhancing walls
	Main duct 5-9 mm
	Abrupt caliber change of main pancreatic duct
	Lymphadenopathy
	Serum level CA 19-9 elevated
	Growth > 3mm/ 12 months; 5mm/24 months
High-risk stigmata	Obstructive jaundice caused by cystic lesions
	Enhancing mural nodule >- 5mm
	Main duct >-10mm

Surveillance protocols vary between guidelines, and no definite protocol is agreed upon for surveillance. However, all guidelines agree that the risk of malignancy, life expectancy, and comorbidities of the patient should be taken into account in the decision-making [36].

In the case of IPMN, for asymptomatic patients without concerning features, surveillance interval is determined by the cyst size according to IAP guidelines, whereas AGA and European guidelines do not consider cyst size. For MCN, AGA and IAP guidelines recommend resection for all cases. On the other hand, European guidelines recommend follow-up in MCN with a size <40mm without mural nodules. Active surveillance is not needed in asymptomatic SCN patients as the risk for malignant transformation is very rare [37].

European Neuroendocrine Tumour Society consensus guideline and European guidelines advise surveillance for patients with asymptomatic cNET tumors of size <20mm. Studies have shown that in asymptomatic tumors <20mm, conservative management is safe [23].

The modality of choice for surveillance is MRI with MRCP. EUS is used in patients who cannot undergo MRI with MRCP. Surveillance is indefinite as long as the patient is surgically fit and willing for surgery if warranted. Table 7 summarises the guidelines on surveillance for cases that don't have at-risk features [38,39].

**Table 7:** Approach to surveillance of pancreatic cysts without high risk or worrisome features [39] IAP (FUKUOKA)- International Association of Pancreatology ACG- American College Gastroenterology ACR- American College of Radiology AGA- American Gastroenterological Association Guidelines EUS- Endoscopic ultrasound MRI- Magnetic Resonance imaging

Size	IAP (FUKUOKA) 2017	ACG 2018	ACR 2018	EUROPEAN 2018	AGA 2015
<1cm	CT/MRI in 6 months then every 2 yearly	MRI every 2 yearly (lengthen after 4 years)	MRI/CT every 1 year for cysts < 1.5 cm and every 6 months for cysts 1.5-2.5 cm × 4 and then lengthen interval. Stop after stability over 10 years	Surveillance every 6 months × 2 with MRI and/or EUS, CA19-9. If stable lifelong surveillance is recommended with annual MRI/EUS, CA19-9	MRI in 1 year then every 2 for 5 years Stop if stable
1-2cm	CT/MRI in 6 months × 1 year Annually × 2 years, then lengthen interval if stable	MRI every 1 year for 3 years then every 2 years for 4 years			
2-3cm	EUS in 3-6 months, then lengthen interval, alternate MRI with EUS as appropriate	EUS/MRI every 6 months for 3 years then yearly for 4 years	For cysts >2.5 cm every 6 months MRI/CT and then stop if stable for over 10 yr. For patients > 80 years of age, q2 year imaging.		
>3cm	Alternate MRI/EUS every 3-6 months	EUS/MRI q 6 mo for 3 years then yearly for four years			

Surgical intervention

Surgical resection of PCN is recommended to be performed by an experienced multidisciplinary team in a high-volume center. Various surgical procedures can be done for PCN depending on the location and size of the cyst; pancreatoduodenectomy is done for PCN located in the head of the uncinate process, whereas distal pancreatectomy is done for PCN in the body or tail. In the neck and proximal body of the pancreas, less extensive resections such as central pancreatectomy can be performed. Central pancreatectomy is a parenchyma-sparing technique that entails removal of the neck and proximal body and sparing the head and tail of the pancreas. However, despite its excellent long-term pancreatic functional outcomes, there is a high risk of pancreatic fistula, thereby increasing the morbidity of the procedure. Hence careful patient selection is of paramount importance [40,41].

IPMN

Resection is indicated in MD-IPMN according to IAP and European guidelines. On the contrary, AGA guidelines only recommend resection if main duct dilatation is present along with a nodule or positive cytology for malignancy. In the case of SB-IPMN, active surveillance or surgical intervention is determined by the presence of malignant features or symptoms. Pancreatic enucleation can also be done in cases of larger SB-IPMN as an alternative to lifelong surveillance. Advantages include decreased pancreatic parenchyma resection and decreased endocrine and exocrine insufficiency. However, in cases of unanticipated malignancy, lack of lymphadenectomy may lead to inferior oncological outcomes.

MCN

Resection is recommended for all lesions according to AGA and IAP guidelines, whereas European guidelines recommend resection of symptomatic MCN if size >-40mm or if a nodule is noted. European guidelines advocate conservative management for MCN <40mm as studies have shown that small-sized asymptomatic indeterminate MCN with no at-risk features have a low risk of malignancy.

SPN

Resection is indicated in patients with SPN according to the AGA, IAP, and European guidelines.

cNET

European guideline and European Neuroendocrine Tumor Society consensus guideline recommend resection of cNETs > 20mm/any malignant behaviour [23].

EUS-Guided Pancreatic Cyst Ablation

Cyst ablation using ethanol or paclitaxel is an experimental approach for PCN. Advantages include organ preservation and preservation of endocrine and exocrine function. Disadvantages include the inability to obtain a specimen for histopathological evaluation [42,43].

Postoperative Surveillance

Patients who underwent resection for MCN do not require surveillance. However, resected MCN with evidence of invasive carcinoma in the histopathological specimen have a 25% risk of recurrence. IPMN cases post partial pancreatectomy must undergo life-long follow-up until the patient is not fit or is unwilling to undergo surgery. European guidelines recommend that follow-up is indicated with cross-sectional imaging every six months for the first two years, followed by yearly imaging for HGD or main duct involvement post resection. IAP guidelines recommend biannual follow-up in patients with a family history of pancreatic malignancy, HGD present in margin, and nonintestinal histological subtype; for the rest, six to 12 monthly follow-up is adequate [44-46].

Recent advances in diagnosis and investigations in PCN

Secretin-Enhanced MRCP

Secretin-enhanced MRCP is used to enhance the visualization of a connection between the PCN and pancreatic duct. It is based on the mechanism of action of secretin which stimulates the acinar cells to release pancreatic juice into the duct, subsequently causing an increase in size and diameter of the duct. The advantages of the use of secretin-enhanced MRCP have been shown in several studies, but long-term studies are yet to be done to justify its use in standard clinical practice. Disadvantages include its cost and increase in scanning time by five to 10 minutes [47].

Contrast-Enhanced EUS

Contrast-enhanced EUS is used to distinguish between mural nodules and clots by determining the vascularity present in mural nodules. It is done by detecting signals from microbubbles in vessels secondary to the contrast agent. Disadvantages are similar to EUS, including its operator dependence and need for specialists experienced in advanced endoscopy [48].

Confocal Laser Endomicroscopy (CLE)

CLE is used as an adjunct to differentiate between PCN types. It uses an endoscopic probe introduced through a 19-gauge needle to display real-time visualization of the PCN with microscopic detail. Characteristic findings for SCN include 'superficial vascular network' or 'fern pattern', whereas for IPMN finger-like papillae are characteristic. Epithelial bands (epithelial layers without a papillary configuration) suggest MCN. Diagnostic accuracy in differentiating between PCN types ranges from 71-94% [49,50].

## Conclusions

Pancreatic cystic neoplasms are being diagnosed at an increased pace primarily due to improvements in radiological and endoscopic imaging modalities. In recent years, numerous guidelines have been created to augment PCN diagnosis, classification and management. Despite this, the management of PCNs remains complex. Thus, discussions with multidisciplinary teams involving surgeons, gastroenterologists, pathologists, and radiologists are required to ensure optimum care to the patient. 
